# Efficacy of a benzothiazole‐based LRRK2 inhibitor in oligodendrocyte precursor cells and in a murine model of multiple sclerosis

**DOI:** 10.1111/cns.14552

**Published:** 2024-01-24

**Authors:** Rocío Benítez‐Fernández, Fernando Josa‐Prado, Estefanía Sánchez, Yolanda Lao, Alfonso García‐Rubia, José Cumella, Ana Martínez, Valle Palomo, Fernando de Castro

**Affiliations:** ^1^ Centro de Investigaciones Biológicas Margarita Salas‐CSIC Madrid Spain; ^2^ Instituto Cajal‐CSIC Madrid Spain; ^3^ Instituto de Química Médica, IQM‐CSIC Madrid Spain; ^4^ Centro de Investigaciones Biomédicas en Red en Enfermedades Neurodegenerativas (CIBERNED), Instituto de Salud Carlos III Madrid Spain; ^5^ Instituto Madrileño de Estudios Avanzados IMDEA Nanociencia Madrid Spain; ^6^ Unidad de Nanobiotecnología Asociada al Centro Nacional de Biotecnología (CNB‐CSIC) Madrid Spain

**Keywords:** LRRK2 inhibitors, multiple sclerosis, remyelinating agents

## Abstract

**Aims:**

Multiple sclerosis (MS) is a chronic neurological disease that currently lacks effective curative treatments. There is a need to find effective therapies, especially to reverse the progressive demyelination and neuronal damage. Oligodendrocytes form the myelin sheath around axons in the central nervous system (CNS) and oligodendrocyte precursor cells (OPCs) undergo mechanisms that enable spontaneously the partial repair of damaged lesions. The aim of this study was to discover small molecules with potential effects in demyelinating diseases, including (re)myelinating properties.

**Methods:**

Recently, it has been shown how LRRK2 inhibition promotes oligodendrogliogenesis and therefore an efficient repair or myelin damaged lesions. Here we explored small molecules inhibiting LRRK2 as potential enhancers of primary OPCs proliferation and differentiation, and their potential impact on the clinical score of experimental autoimmune encephalomyelitys (EAE) mice, a validated model of the most frequent clinical form of MS, relapsing–remitting MS.

**Results:**

One of the LRRK2 inhibitors presented in this study promoted the proliferation and differentiation of OPC primary cultures. When tested in the EAE murine model of MS, it exerted a statistically significant reduction of the clinical burden of the animals, and histological evidence revealed how the treated animals presented a reduced lesion area in the spinal cord.

**Conclusions:**

For the first time, a small molecule with LRRK2 inhibition properties presented (re)myelinating properties in primary OPCs cultures and potentially in the in vivo murine model. This study provides an in vivo proof of concept for a LRRK2 inhibitor, confirming its potential for the treatment of MS.

## Graphical abstract

Methodology of selection of a LRRK2 inhibitor effective in OPC proliferation, differentiation, and in a murine model of multiple sclerosis.

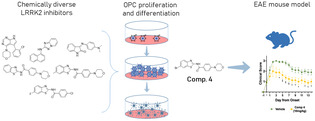

## 1 INTRODUCTION

Multiple sclerosis (MS) is a common demyelinating disease that is diagnosed in young adults (20–40 years of age) with symptoms that range from fatigue, mobility problems, muscle spasms, and pain. Most of the patients are diagnosed with a relapsing–remitting variety of the disease in which neurological symptoms are totally or partially recovered after each relapse (RR‐MS). During the course of the disease, a significant proportion of patients (up to 80%) develop a secondary progressive form of the disease in which neurological symptoms and disability will steadily increase with or without the appearance of relapses (SP‐MS).^1^ While several therapeutic options have been approved for MS in the last years, there is a need to find drugs capable of modifying the demyelinating pathological cascades that occur during the first stages of the disease and promote spontaneous remyelination.^2^

Regarding its molecular characteristics, MS is a chronic inflammatory, autoimmune, and neurodegenerative disease, characterized by glial cell pathology (especially oligodendrocytes and their precursors), demyelination, inflammatory processes, and axonal damage in the central nervous system (CNS). The cause/s of the disease are not known, however the lesions occurring in the brain are associated with the activation of T and B lymphocytes that target autoantigens in several structures of the CNS.^3^ In these lesions, the cells specialized in the formation and maintenance of myelin, and in providing nurturing support to neurons, die, forming the demyelinating plaque. With time, axons become affected and neuronal death can even be achieved. After myelin damage, the spontaneous activation of remyelination occurs in order to preserve the integrity of affected neurons.^4^ This fundamental process is attributed to oligodendrocyte precursor cells (OPCs) that proliferate, migrate to the lesion, and differentiate into mature oligodendrocytes. However, the chronic inflammation and toxic environment in MS brains hinder the successful execution of this repairing process, which is not sufficient to stop the progression of the disease. For these reasons, promoting this endogenous repairing mechanism is a promising therapeutic strategy for the treatment of MS.^5^ There are several successful examples of this strategy, including the inhibition of MAPK/ERK that enhances this repairing process in several models of MS, or the inhibition of PDE7, that has shown to promote OPC proliferation and differentiation to mature oligodendrocytes in vitro and in vivo.^6^, ^7^ In addition, the first proof of concept of remyelination in human by small molecules was achieved in 2017 with the first clinical trial with clemastine fumarate, where a remyelinating effect was observed independently from immunemodulation.^8^ Currently, a number of small molecules targeting remyelination can be found at preclinical and clinical stages, including benztropine, GSK‐3 and PDE7 inhibitors and H3 receptor antagonists.^9^ These advances, together with the new methodologies for remyelination quantification,^10^ highlight the potential of compounds with remyelinating properties, which especially in combination with anti‐inflammatory therapies could lead to an improved therapeutic option for MS patients.^11^

Leucine‐rich repeat kinase 2 (LRRK2) is a large cytosolic kinase classified in the Roco protein superfamily and adopts multiple functions and domains.^12^, ^13^ The discovery of LRKK2 gene as a major susceptibility factor for Parkinson's disease in 2004,^14^, ^15^ activated the research in this area as a potential mechanism to therapeutically target this disease. Recently, there have been several associations of this enzyme to processes of inflammation that could contribute to Parkinson's or other diseases, including MS.^16^ In addition, LRKK2 may contribute not only to inflammation processes but also to neurotoxic processes involved in neurodegenerative disorders^17^ and thus may be a key player in the progression of neurodegenerative pathologies.^18^ The relevance of LRRK2 for neurogenesis in the adult CNS, neurite outgrowth, and the formation of axons and dendrites is also known.^19^, ^20^ In addition, LRRK2 has been found to be expressed in several neural cell populations, including human oligodendroglia^21^ and OPCs.^22^

Several LRKK2 inhibitors have been discovered due to its potential to treat Parkinson's disease.^14^, ^15^, ^23^ The first generation of inhibitors consisted of non‐selective Type I kinase inhibitors^24^ that evolved to more selective agents belonging to the family of aminopyrymidynes.^25^, ^26^ Our group discovered two families of compounds that inhibit LRRK2 and have shown to have neurogenic properties. First, a family of indolinones with selective micromolar IC_50_ activities^27^ and more recently, a family of benzothiazoles with submicromolar selective inhibition of LRRK2.^28^ Interestingly, both families exerted a neurogenic behavior promoting the proliferation of neural stem cells and the benzothiazoles were able to stimulate their differentiation to the oligodendrocytic lineage. Given these promising properties, in this work, we aimed to explore the potential of these families of compounds to treat MS.

## 2 MATERIALS AND METHODS

### 2.1 Drugs

The inhibitor PF‐06447475 (PF‐475) was obtained from Sigma‐Aldrich. Compound **1** was prepared using the synthetic procedures described in the Appendix S1. Compounds **2–5** were synthesized following a previously published synthetic route.^27^, ^28^

### 2.2 Animals

Postnatal P0‐P3 Wistar rats, bred at the Instituto Cajal‐CSIC, were used for isolating OPCs for cultures. C57 mice were obtained from Charles River Laboratories (Wilmington, MA, USA) and maintained in the animal facilities at Instituto Cajal (Madrid, Spain). Adult mice were sacrificed for histological procedures after inducing the experimental autoimmune encephalomyelitys (EAE) model (see below). All procedures were approved by CSIC's institutional ethics committee (440/2016, Madrid, Spain) and followed the Spanish (RD 53/2013, 178/2004, Ley 32/2007, Ley 9/2003, and RD 320/2010) and European regulations (2010/63/EU, 90/219/EEC).

### 2.3 Dissection and tissue dissociation of rat cortices

As previously described^6^, ^29^ the whole rat brains were harvested from ice‐anesthetized postnatal day 0–3 pups by decapitation following loss of reflexive movement (approximately 5–8 min in ice). The skin and skull were cut with a scalpel along the midline and reflected to expose the brain. The brain was then carefully removed and placed in a Petri‐dish with sufficient volume of HBSS with Ca^2+^ and Mg^2+^ (at 4°C) to submerge the brain. The Petri‐dish was kept on ice for the rest of the procedure. Afterward, the cerebellum, brainstem, olfactory bulbs, and olfactory tracts were removed, and the meninges and the hippocampus were carefully removed from the brain. Finally, all dissected cortices were transferred into a 50 mL Falcon tube with HBSS without Ca^2+^ and Mg^2+^ (HBSS−/−). For the tissue dissociation, the papain‐based Neural Tissue Dissociation Kit (Miltenyi Biotec, Bergisch Gladbach, Germany) was used. Due to proprietary reasons, Miltenyi was unable to provide details on the specific composition of their reagents. Using the Neural Tissue Dissociation Kit (Miltenyi Biotec), an appropriate volume of Enzyme Mix 1 corresponding to the cortical tissue mass was prepared. For 4–5 cortices, 50 μL of Enzyme P was added to 1.9 mL of buffer X. This was briefly vortexed and then preheated in a 37°C water bath for 10 min before use. As Enzyme Mix 1 was heating, the neural tissue was transferred to a Petri dish, where they were diced thoroughly with a blade. Then HBSS−/− was added and tissue was collected in a 15 mL falcon, and let stand for 2 min to allow the tissue to drop and the remaining meninges to remain in the supernatant for removal by aspiration. HBSS−/− was added and tissue was centrifuged at 300 *g* for 2 min at room temperature (RT). The supernatant was then carefully aspirated, followed by the addition of the pre‐heated Enzyme Mix 1 to the pellet, and then gently mixed taking care to avoid air bubbles. Tissue was then incubated for 15 min in 37°C under slow and continuous rotation. In the meantime, Enzyme Mix 2 was prepared adding 10 μL of Enzyme A to 20 μL of buffer Y. After incubation, Enzyme Mix 2 was added to the tube containing the tissue. This was inverted gently to mix and then slowly triturated approximately 15 times using 5 mL pipette taking care to avoid air bubbles. Then, this was incubated for 10 min in the 37°C under slow and continuous rotation (50 rpm). Tissue was slowly disaggregated by pipetting up‐and‐down approximately 15–20 times each using a 5 mL pipette. Then tissue was incubated again for 10 min in 37°C under slow and continuous rotation (50 rpm). During the incubation, a 70 μm cell strainer was pre‐wetted with 500 μL Miltenyi washing buffer (MWB) into a 50 mL falcon. The 15 mL tube with the tissue was rinsed with 10 mL of HBSS−/− and added to the strainer. Before centrifugation, the total number of live cells was counted by Trypan Blue visualization. The cell suspension was centrifuged at 300 *g* for 10 min at RT.

### 2.4 Magnetic cell sorting for OPCs isolation

As previously reported,^29^, ^30^ following the last centrifugation, supernatant was gently aspirated and the pellet was resuspended in primary antibody solution (4 μL A2B5 antibody in 1000 μL MWB per 10^6^ cells) and the incubation was for 25 min at 4°C under slow shaking. Later, 1–2 mL MWB was added and cell suspension was centrifuged at 300 *g* for 10 min at RT. The supernatant was gently aspirated and the pellet was resuspended in secondary antibody solution (40 μL A2B5 antibody in 160 μL MWB per 10^6^ cells) and incubated for 15 min at 4°C under slow shaking. After incubation, 1–2 mL MWB was added and cell suspension was centrifuged at 300 × g for 10 min at RT. During centrifugation, we set up a magnetic cell sorting (MACS) MS column on a MACS magnetic stand (Miltenyi Biotec) and equilibrated the column 500 μL MWB. When the centrifugation was finished, cells were suspended in 1000 μL MWB and then added carefully in the MS column, which was washed three times with 500 μL MWB. Quickly, the magnet was removed, column was placed on a 15 mL falcon, 1000 mL of OPCs medium (Neurobrew‐21, 0.5% P/S, 1% glutamine, 0.05% PDGFα, 0.05% FGF2, 20 mM glucose, Neuromedium) was added and plunger was push. Cells were counted, 50 μL of cell solution was plated in a poli L‐lysine/laminin‐coated 12 μm coverslip in a 24 well plate or a 96‐well plate and incubated in 37°C, 5% CO_2_ for 1 h. Later, each well was added with 500 μL OPCs medium (with growth factors). Approximately, 30,000 cells/well were plated in a 24 well plate and 15,000 cells/well in a 96 well plate for a successful OPC isolation. The primary cultures used for the present study had a minimum of 95% purity.

### 2.5 OPCs proliferation and differentiation

The day following OPC isolation, viability assay, and proliferation assay were done proliferating cells with an OPCs medium.^29^, ^30^ The composition of the medium was: 0.5% Neurobrew‐21 (Miltenyi Biotec), 0.6% penicillin/streptomycin, 1% glutamine, 0.01% PDGF‐AA, 0.01% FGF‐2, 2% glucose and all dissolved in MACS® Neuro Medium. Proliferation assay was performed in 24‐well plates, where LRRK2 inhibitors to be tested and BrdU were added at the same time and incubated for 6 h in 37°C, 5% CO_2_. Then, BrdU was removed and LRRK2 inhibitors were incubated overnight in OPCs medium (with growth factors) in 37°C, 5% CO_2_. For the differentiation assay, all medium was removed from the plate and replaced with fresh OPCs medium. The day after, the medium was removed and the cells were incubated with OPCs medium without growth factors (PDGFα and FGF2) for 24 h to allow cells to differentiate. Then all the inhibitors were added and incubated in the same medium for 48 h in 37°C, 5% CO_2_ without growth factors to allow cells to differentiate. In this assay, T3 is a thyroid hormone that was used as a positive control for differentiation because this hormone stimulates oligodendroglial differentiation.^31^, ^32^ T3 was used at 30 ng/mL final concentration in the culture medium. Data were first tested for normality and indicated in each of the cases together with the statistical methodology performed.

### 2.6 Viability assay

Viability assay was performed in 96‐well plates, where all inhibitors to be tested and H_2_O_2_ at 10 μM as internal control were added and incubated in OPCs medium for 24 h in 37°C, 5% CO_2_. The day after, we added 10 μL of 3‐(4,5‐dimethylthiazol‐2‐yl)‐2,5‐diphenyl‐2H‐tetrazolium bromide (MTT) (Sigma‐Aldrich) at 5 mg/mL per well and incubated for at least 4 h. The medium was aspirated and added 200 μL of DMSO (Scharlau) to dissolve the formazan formed. Finally, results were obtained on the FLUOstar OPTIMA (BMG Labtech) at 595 nm. Data were tested for normality with the D'Agostino & Pearson test.

### 2.7 Immunocytochemistry

For the proliferation assay, the medium was aspirated and plate rinsed with sterile PBS (1×). Cells were fixed with Paraformaldehyde (PFA) (4% in PB) for 10 min, then washed with PBS (1×) for three times for 10 min, incubated 1 h at RT with blocking buffer [PBS ‐1×‐, 5% normal donkey serum (NDS)] and incubated with primary antibody (Table 1) at 4°C overnight in the humidity chamber. The following day, cells were washed three times for 10 min with PBS (1×) under shaking and were incubated with the secondary antibody for 1 h at RT with shaking. Then, the cells were washed three times for 10 min with PBS (1×) under shaking and fixed with previously tempered PFA (4%) for 10 min under shaking. Again, cells were washed three times for 10 min with PBS (1×) under shaking, denatured with HCl (2 N) for 45 min at RT, washed three times for 10 min with PBS (1×) under shaking and washed three times for 10 min with Borate Buffer pH 8.5. Next, cells were washed three times for 10 min with 1× PBS under shaking and incubated for 3 h with the blocking buffer (PBS ‐1×‐, FBS 5%, Triton 0.3%, gelatin 0.2%, glycine 0.2 M). Then, cells were incubated with the primary antibody against BrdU at 4°C overnight. In the last day of the procedure, cells were washed three times for 10 min with 1× PBS under shaking and incubated with secondary antibody for 2 h at RT with shaking, then washed three times for 10 min with PBS (1×) under shaking and incubated with Hoechst (1:10) in PBS (1×) for 10 min in the humidity chamber. After the last wash with PBS 1×, and another wash with H_2_O mQ to remove any PBS (1×), the coverslips were mounted with ImmunoMount®. Data were tested for normality with the Shapiro–Wilk test.

**TABLE 1:** List of antibodies used in this study.

Antibody	Target	Cellular location	Dilution	Host species	Class	Manufacturer	ID
MBP	Myelin	Plasma membrane	1:500	Rat	Monoclonalclone 12	Biorad	aa 82–87
BrdU	Proliferation Marker	Nucleus	1:1000	Rat	Monoclonal	Abcam	ab6326
CC1	Mature oligodendrocytes	Cell body	1:200	Mouse	Monoclonal clone CC1	Merck Millipore	OP 80
Olig2	Oligodendrocyte lineage	Nucleus	1:200	Rabbit	Polyclonal	Merck Millipore	AB 9610
Iba1	Microglia	Cytoplasm	1:500	Guinea Pig	Polyclonal	Synaptic Systems	234,004
A2B5	OPCs marker	Plasma membrane	1:200	Mouse	Monoclonal	Merck Millipore	MAB312

Abbreviations: MBP, myelin binding protein; NFH, neurofilament heavy polypeptide; PDGFRα, platelet‐derived growth factor receptor α.

For the differentiation assay, all medium was aspirated and the plate was rinsed with sterile PBS 1× and cells were fixed with PFA (4%) for 10 min, then washed with PBS 1× for three times for 10 min and incubated 1 h at RT with blocking buffer (PBS ‐1×‐, NDS ‐5%‐). Incubated with primary antibody (Table 1) at 4°C overnight in the humidity chamber. The following day, cells were washed three times for 10 min with 1× PBS under shaking. Then, cells were incubated with the corresponding fluorescent (1:1000; Invitrogen, Paisley, UK) secondary antibodies for 1 h at RT with shaking, washed three times for 10 min with PBS (1×) under shaking and incubated with Hoechst 1 μg/mL in PBS (1×) for 10 min in the humidity chamber. After the last wash with PBS (1×), and another wash with Milli Q H_2_O to remove any PBS (1×), the coverslips were mounted with ImmunoMount®. Differentiation is measured as a significant increase in the number of OPCs that became oligodendrocytes (MBP^+^Olig2^+^/Olig2^+^) relative to the total number of Olig2 cells in the cultures, compared with the control. Data were tested for normality with the Shapiro–Wilk test.

In all cases, a one‐way ANOVA analysis was performed. In the event of not finding statistical significance, a Student's *t*‐test was performed to compare the control and each of the treatments, as indicated in each figure caption.

### 2.8 Experimental autoimmune encephalomyelitis (EAE) model

We used the EAE murine model, which is widely used to study the autoimmune aspects of MS.^33^, ^34^, ^35^ This model involves the auto‐immune processes that ends in a demyelinating process along the CNS to induce in mice a MS model that involves autoimmune response. In short, at Day 0, 6‐ to 8‐week‐old C57Bl6 female mice were anesthetized with xylazine (20 mg/mL) and ketamine (37 mg/kg). Then they were immunized with the fragment peptide 35–55 of the MOG protein (M‐Q‐V‐G‐W‐Y‐A‐S‐P‐F‐S‐ A‐V‐V‐H‐LY‐A‐N‐G‐K) (Genscript HK Limited ‐Hong Kong‐) in PBS (250 μg/100 μL × mouse) emulsified with Freund's Complete Adjuvant (100 μL/mouse) [a mixture of Freund's Adjuvant Incomplete (Sigma‐Aldrich) and inactivated Mycobacterium tuberculosis particles ‐H37RA Difco‐ at 4 mg/mL final concentration] by repeated alternating flows between two syringes through a 3‐way stopcock. Mice were subcutaneously injected with this emulsion near both axillary and both inguinal lymph nodes (2 + 2 injections, 50 μL each). To induce opening of the blood–brain barrier for facilitating leukocyte migration to the central nervous system, 100 μL/mouse of pertussis toxin (4 ng/μL; Sigma‐Aldrich) were subsequently injected intraperitoneally. At day 2, mice were injected with a second dose of pertussis toxin in the same manner.

Weights and clinical scores were registered daily for each mouse using a scale from 0 to 5, adapted from previously described scoring in literature.^34^, ^36^ EAE was double‐blindly scored as follows: Clinical scores: 0, no symptomatic signs; 0.5, limp distal tail (flaccidity‐absence of curling); 1, limp tail (whole) or hind limb weakness but not both; 1.5, limp tail (whole) and one hind limb weakness; 2, limp tail and both hind limbs weakness; 2.5, limp tail and dragging hind limbs with little responsiveness/movement or complete paralysis of one hind limb; 3, limp tail and complete paralysis of both hind limbs (dragging) or minimal paddling with affection of one forelimb; 3.5, limp tail and complete paralysis of both hind limbs (dragging) and partial paralysis of one forelimb; 4, limp tail and complete paralysis of both hind limbs (dragging) and partial paralysis of both forelimbs; 4.5, little to no movement, unresponsiveness; 5, moribund (euthanasia) or found dead. Cumulative values have been calculated for each day, as the aggregation of the clinical score value of a given day to the sum of the values of the previous days. Data were tested for normality with the Shapiro–Wilk test.

### 2.9 In vivo treatment of EAE mice and perfusion of the animals

Compound **4** was suspended in vehicle solution (Saline Serum with 5% DMSO and 1% TWEEN‐20) and sonicated before use. Once mice showed the onset of the EAE (0.5 or more of clinical score) they started receiving a daily intraperitoneal injection of either vehicle as control or compound **4** at 10 mg/kg. Mice were kept up until day 14 post‐onset and then, animals were euthanized and their tissues fixed by intracardiac perfusion with 4% PFA in phosphate buffer (PB). After perfusion, the brain and spinal cord were carefully dissected, separately removed, and immersed in PFA (4%) for 24 h, then cryo‐protected with incremental concentration of sucrose up until 30% in PB, and finally included in OCT (QPATH, Thermo Scientific) for cryo‐preservation. Upper thoracic spinal cord was sectioned (20 μm thickness, in a transversal plane), placed onto Superfrost® Plus histological slides (Thermo Scientific), and frozen at −20°C until used for histological and immunocytochemistry procedures.

## 3 HISTOLOGY AND IMMUNOCYTOCHEMISTRY

For immunohistochemical stainings, the tissue cryosections were thawed and let to air‐dry, then blocked with the blocking solution (PBS ‐1×‐, 5% NDS, 0.2% TritonX‐100) for 1 h at RT in a humidity chamber. Then, antibody anti‐Iba1 was diluted in the blocking solution and used to cover the tissue over/night at RT in humid chambers. After three washes in PBS (1×), the fluorophore‐tagged secondary antibody in blocking solution was added onto the tissue for 2 h at RT in a humidity chamber. Finally, commercial Fluoromyelin‐Red solution (1:3000, Invitrogen, F34652) and Hoechst (1 mg/mL in PBS ‐1×‐) were added and incubated in a humidity chamber for 20 min prior to the final washing (3 times in PBS ‐1×‐) and mounting in Fluoromount‐G™ (Invitrogen).

For myelin staining, the classical eriochrome‐cyanine method was used as previously described.^30^, ^34^ In brief, sections of tissue, adjacent to those immunostained, were dried for 2 h at RT, and then for 1.5 h at 37°C. The slides were then placed in a container with acetone for 5 min at RT, and then air‐dried for 30 min. The sections were stained in eriochrome‐cyanine solution [Solution 1 (100 mL) + Solution 2 (100 mL); Solution 1: 0.2% eriochrome cyanine RS (Sigma‐Aldrich) and 0.5% v/v of concentrated H_2_SO_4_ in H_2_O; Solution 2: 10% of ferric ammonium sulfate (Sigma‐Aldrich) in H_2_O] for 30 min. Then, to reveal the gray matter, sections were sequentially differentiated in 5% ferric ammonium sulfate (in H_2_O) and 1%borax‐1.25%ferricyanide (in H_2_O) for 10 and 5 min, respectively, briefly rinsing under tap and distilled water between each step. Myelin will remain stained in blue while non‐myelinated areas will be washed out to a white‐ochre color. Data were tested for normality with the Shapiro–Wilk test.

## 4 RESULTS

### 4.1 Effect of LRRK2 inhibitors in OPCs culture viability

**FIGURE 1:**
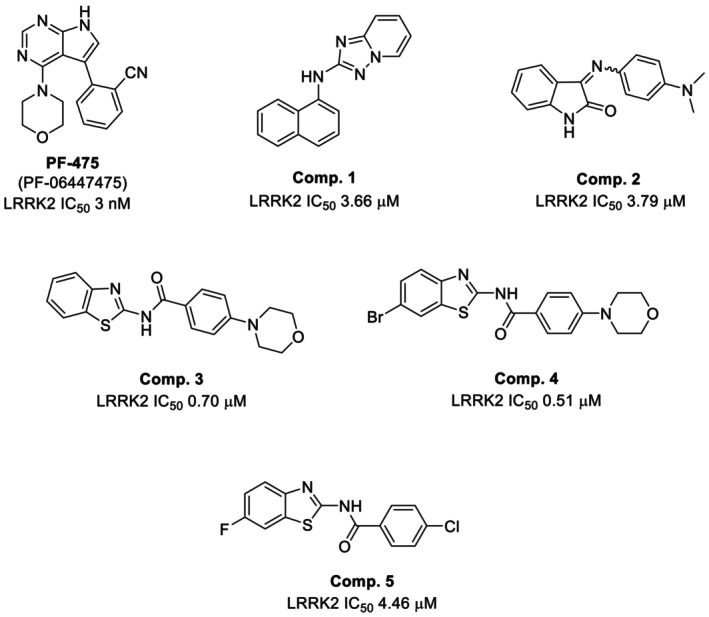
Structure of chemically diverse LRRK‐2 inhibitors selected to study their oligodendrogenic effect. IC_50_ values of LRRK2 are shown below each structure.

In order to assess the oligodendrogenic effect of LRRK2 inhibitors in primary OPCs cultures, we selected a chemically diverse set of antagonists of this enzyme (Figure 1). This strategy enabled us to validate the target effect through the chemical genetic approach. We included the well‐known LRKK2 inhibitor known as PF‐06447475 (PF‐475)^12^ together with some heterocyclic inhibitors either prepared specifically for this work, or already known in our group that had previously shown to favor the differentiation of neurospheres isolated from the subventricular zone of adult mice toward oligodendrocytes.^27^, ^28^

**FIGURE 2:**
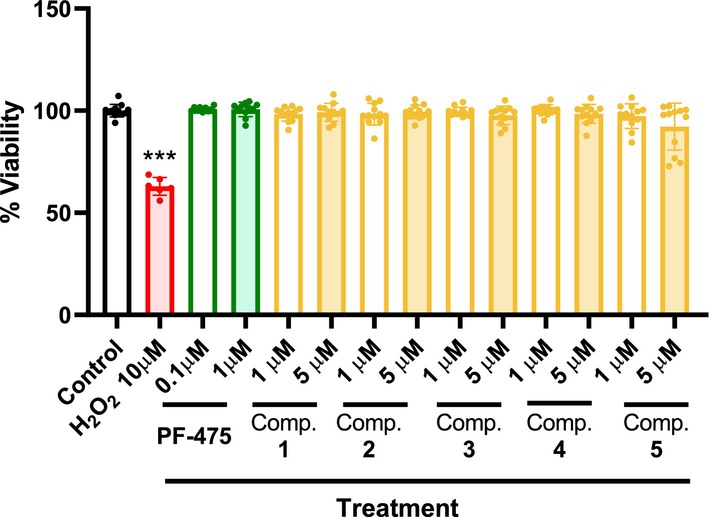
Cell viability. OPCs cultures were treated for 24 h with six chemically diverse LRRK2 inhibitors at two different concentrations and cell viability was measured. Values represent the mean ± SEM of three replications in three different experiments. Statistical analysis by one‐way ANOVA followed by Dunnet's post hoc test is represented as ***p < 0.001 versus control.

Prior to determining the potential efficacy of these compounds, we evaluated their safety in vitro. OPCs viability was checked using the 3‐[4,5‐dimethylthiazol‐2‐yl]‐2,5‐diphenyltetrazolium bromide (MTT) test at two different concentrations of the compounds for 24 h. We selected 1 and 5 μM for the inhibitors (**1**–**5**) given their micromolar IC_50_ activities (Figure 1), and 0.1 and 1 μM for the nanomolar commercially available LRRK2 inhibitor PF‐475 used as reference. As a positive control for toxicity, we used H_2_O_2_. H_2_O_2_ is stable in abiotic environments at ambient temperature and neutral pH, yet rapidly kills any type of cells by producing highly reactive hydroxyl radicals. We observed that in all cases, both concentrations used of the inhibitors did not affect the cell survival, as can be seen from the viability calculated in Figure 2.

### 4.2 LRRK2 inhibitors promote the proliferation of OPCs

**FIGURE 3:**
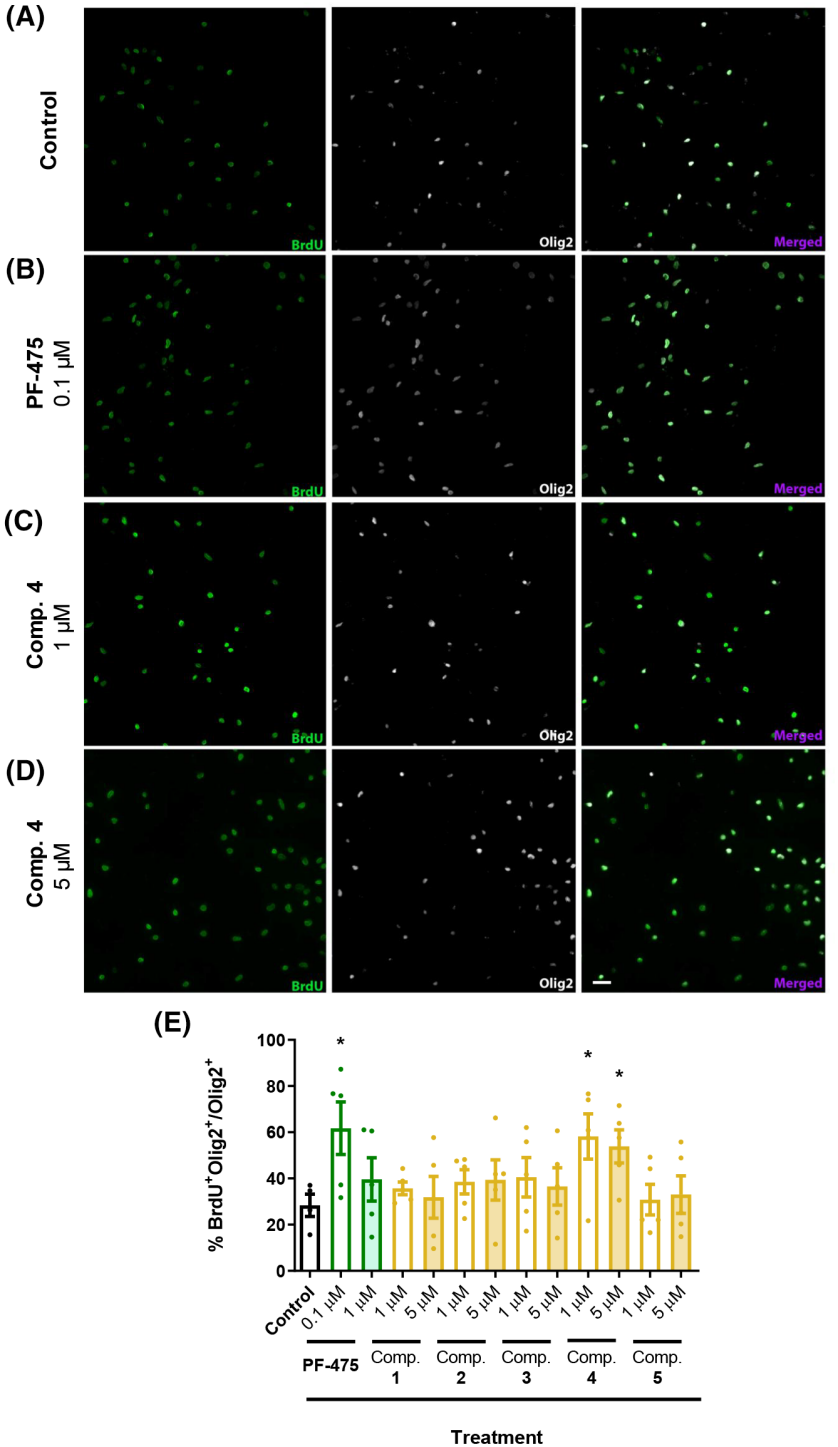
The inhibition of LRRK2 by PF‐475 and compound **4** promotes the proliferation of OPCs from rat brain cortices. (A) Representative images of OPCs cultures treated for 24 h with PF‐475 (0.1 μM) and **4** (1 and 5 μM) 6 h with BrdU, the number of BrdU^+^Olig2^+^ was measured. (B) Quantification of proliferating OPCs (BrdU^+^/Olig2^+^double staining) was performed in respect to the total number of oligodendroglial cells (Olig2^+^) for LRRK2 inhibitors PF‐475 and compounds 1–5. Values represent the mean ± SEM of three replications in five different experiments. Results of Student's *t*‐test are represented as: *p < 0.05 versus control. The scale bar represents 25 μm in A–D.

As no concentration of each inhibitor did affect cell survival (Figure 2), all six LRRK2 inhibitors were selected for the proliferation experiment. OPCs were cultured for 1 day in the presence of inhibitors and their corresponding control, and in the presence of BrdU (see Section 2). The proliferation of OPCs was measured as the number of OPCs incorporating BrdU (BrdU^+^Olig2^+^/Olig2^+^) relative to the total number of oligodendroglial cells (Olig2^+^). Among all inhibitors, only PF‐475 and LRRK2 inhibitor **4** showed an increase in OPC proliferation: while PF‐475 significantly promoted BrdU incorporation only at 0.1 μM (Figure 3A,B,E), LRRK2 inhibitor **4** had a significant effect on OPC proliferation at both 1 μM and at 5 μM (Figure 3A,C–E). Compounds **1**–**3** and **5** did not promote the proliferation of OPCs either at 1 or 5 μM.

#### 4.2.1 LRRK2 inhibitors stimulate OPC differentiation

**FIGURE 4:**
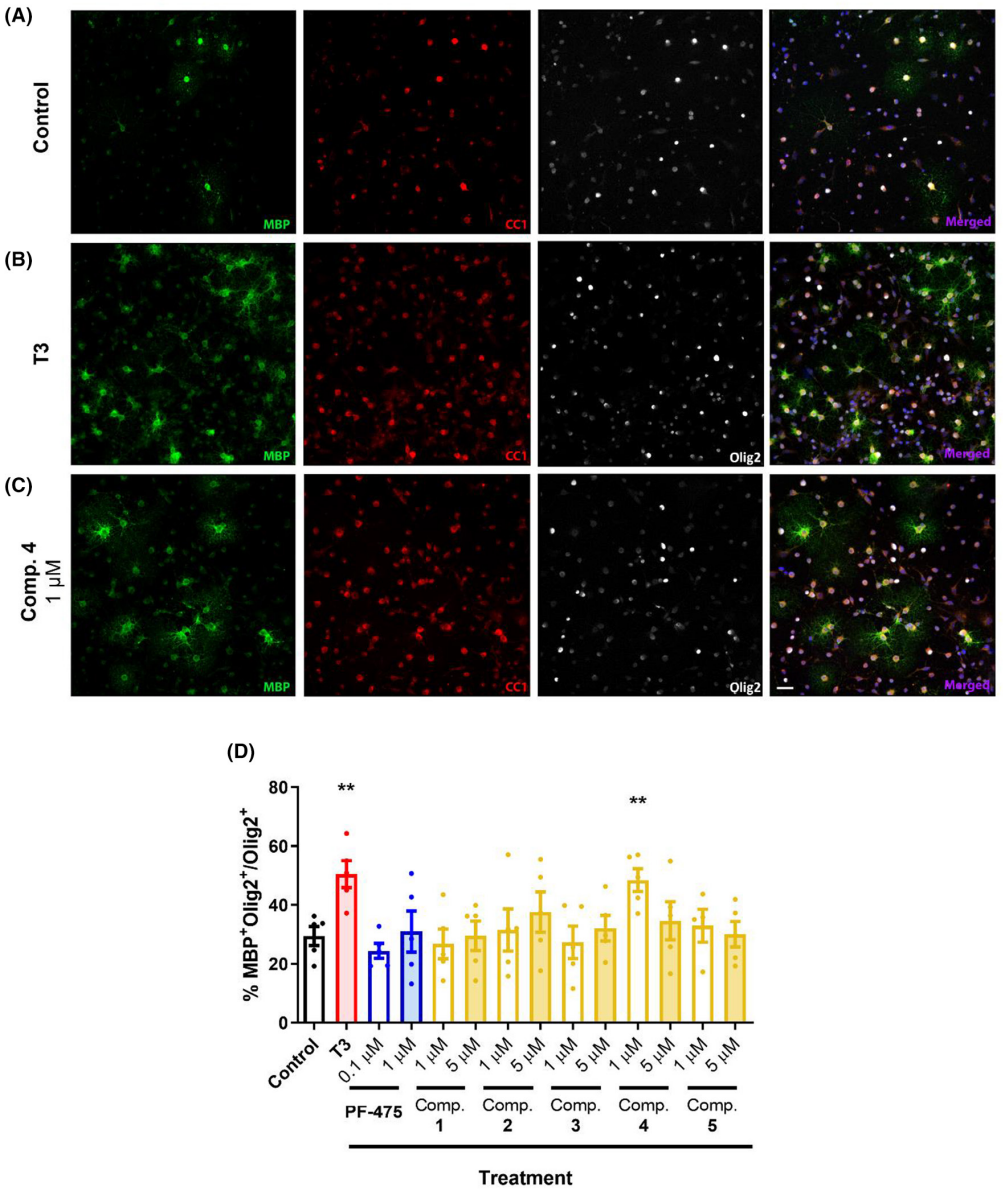
LRRK2 inhibitor **4** increases the differentiation of OPCs from P0‐P3 rat brain cortices. (A) Representative images of oligodendrocytes cultures treated for 48 h with LRRK2 inhibitors (at a concentration of 1 and 5 μM). Oligodendrocyte cultures were stained with Olig2 (the entire oligodendroglial lineage), CC1 and MBP (both typical of myelin‐forming mature oligodendrocytes). (B) Quantification of oligodendrocytes (MBP^+^Olig2^+^) in respect to the total number of oligodendroglial cells (Olig2^+^). Values represent the mean ± SEM of three replications in five different experiments. Results of Student's *t*‐test are represented as: **p < 0.01 versus control. The scale bar represents 25 μm in A–C.

To explore the effect on OPC differentiation, we used OPCs cultured for 2 days in the presence of all inhibitors at the same concentrations selected previously. In addition, thyroid hormone T3 was used as a positive internal control for the experiment at 30 ng/mL concentration, the known dose to promote OPC differentiation.^37^ Myelin‐forming phenotypes were identified as double‐stained for MBP and Olig2 and the ratio of MBP^+^/Olig2^+^ cells per total Olig2^+^ cell was also calculated. Results showed that only T3 (Figure 4A‐B, D) and compound **4** at 1 μM significantly increased proliferation (Figure 4A, C‐D), but none of the other inhibitors at this concentration nor any at 5 μM (Figure 4D).

### 4.3 EFFECT OF COMPOUND **4** IN THE EAE IN VIVO MODEL OF MULTIPLE SCLEROSIS

Based on the studies in vitro on the proliferation and differentiation of OPCs, we decided to check the effects of LRRK2 inhibitor **4** in vivo, using the experimental autoimmune encephalomyelitys (EAE) murine model of MS. We studied the effect of this drug and selected a dose of 10 mg/kg given its LRKK2 submicromolar IC_50_ in vitro. The drug was injected intraperitoneally to EAE‐induced mice daily once they showed EAE onset (≥0.5 clinical score) for 14 days and its effects were compared to the injection of the vehicle.

**FIGURE 5:**
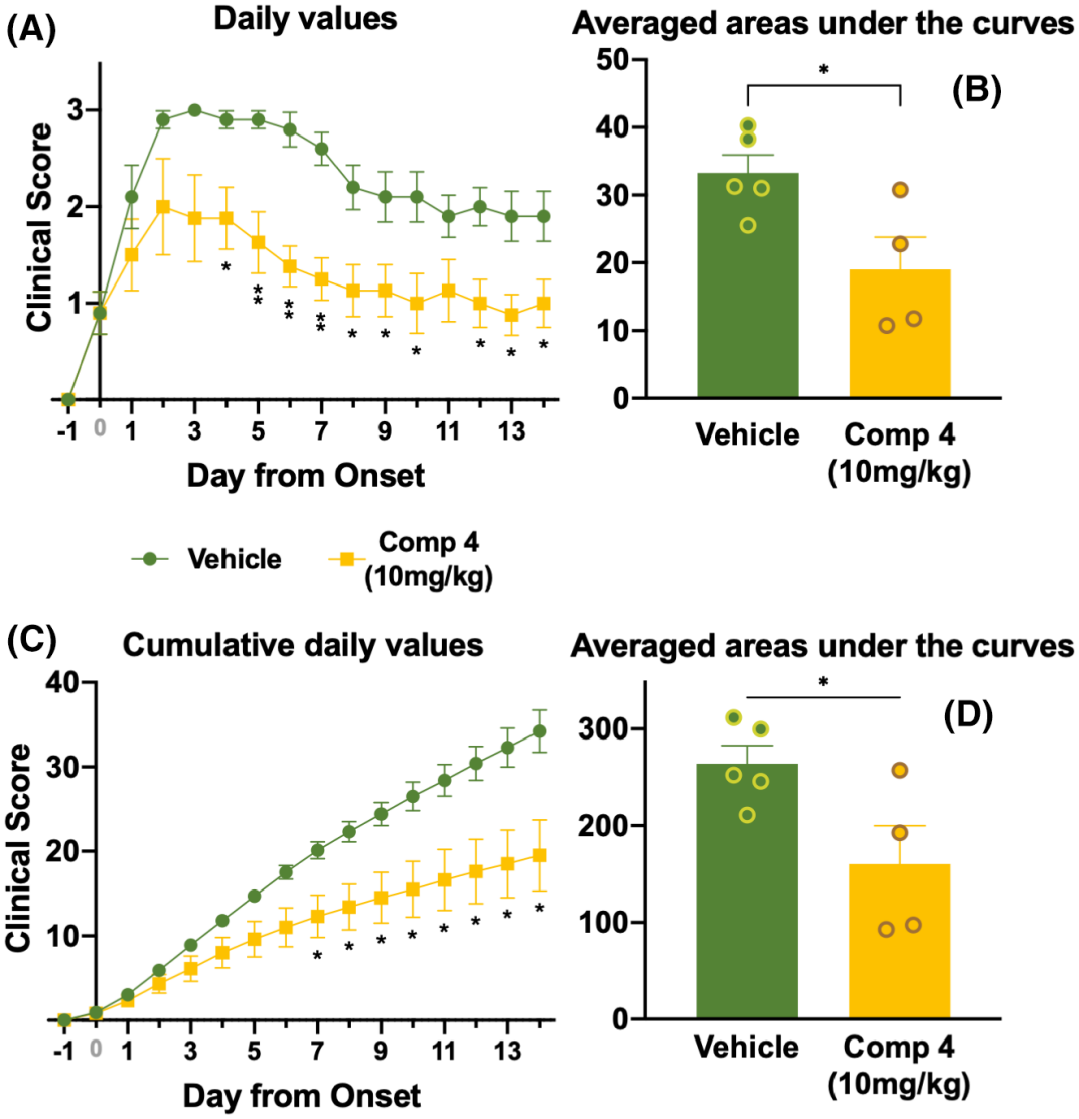
Clinical score results for compound **4** treatment of EAE‐induced mice. Daily vehicle (*n* = 5, dark green) or 10 mg/kg compound **4** (*n* = 4, yellow) were intraperitoneally injected from clinical onset (considered as the first clinical score ≥0.5) for 14 days. (A) average daily values for clinical score and (B) its averaged areas under each mice's clinical score curves. (C) average cumulative daily for clinical score and (D) its averaged areas under each mice's cumulative clinical score curve. Graphs represent average and SEM. Differences between vehicle and compound **4**, both for intra‐day score values (A, C) as well as for averaged values under the curves (B, D), were analyzed by Student's *t*‐test and represented as *p < 0.05, **p < 0.01.

LRRK2 inhibitor **4** ameliorated the clinical score of the disease both in the daily comparative chart (Figure 5A,B), and although the treatment systematically diminished the clinical score from the day moment, results were significant from the peak phase of disease (~2 reached when treated with compound **4** vs. ~3 clinical score reached when treated just with vehicle) onwards. In the cumulative values chart, treatment with compound **4** was significantly effective by day 7 after onset (Figure 5C,D).

### 4.4 Effect of LRRK2 inhibitor **4** on inflammatory response and CNS myelin

**FIGURE 6:**
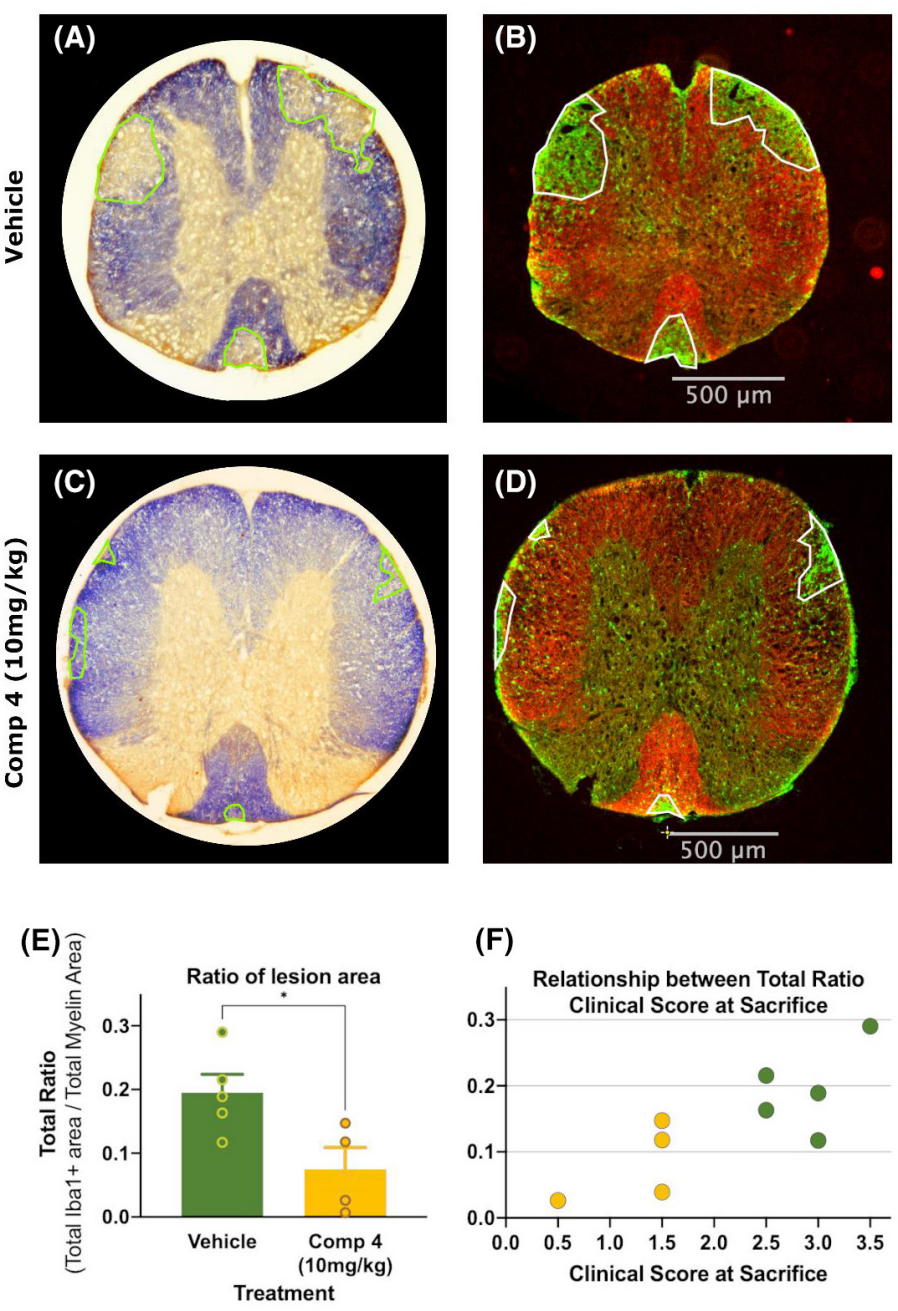
Analysis of lesion area for compound **4** treatment of EAE‐induced mice. (A, B) Spinal cord sections of mice treated with Vehicle (*n* = 5, dark green). (C, D) Cuts of T1 segment of mice treated with compound **4** 10 mg/kg (*n* = 4, yellow). A and C stained with the eriochrome‐cyanine procedure; B and D immunostained against Iba1 (green) with Fluoromyelin‐red stain (red). (E) averages and SEMs of the ratios of the Iba1+ stained area to total myelinated area for Vehicle and compound **4** at 10 mg/kg. Differences were analyzed by Student's *t*‐test and represented as: (*p < 0.05). **F**: ratios of the Iba1+ stained area to total myelinated area plotted against the Clinical Score at sacrifice.

Eriochrome cyanine (Figure 6A,C) and Iba1 plus Fluoromyelin Red (Figure 6B,C) stainings were performed to in order to assess the white matter impairment under vehicle (Figure 6A,B) or treatment with compound **4** (Figure 6C,D). Lesion areas were quantified as the total area of Iba1 and the loss of eriochrome cyanine staining and ratioed against total white matter area (Fluormyelin Red). The ratio of Iba1+ lesion area to total myelin area in the compound **4** treated mice (0.075 ± 0.034) was significantly reduced compared to the mice receiving just the vehicle (0.195 ± 0.029; Figure 6E). Interestingly, vehicle‐treated EAE mice almost systematically scored higher than the compound **4** treated ones (Figure 6E). When the individual ratios for each animal were confronted to their clinical score at the moment of sacrifice, the experimental cases were clearly separated into two groups corresponding to the two different treatments, with the vehicle group occupying the higher‐ratio/higher‐score area of the graph and the treated group occupying the lower‐ratio/lower score area of the graph (Figure 6F). These results, together with the clinical score differences shown above (Figure 5), indicate that compound **4** ameliorates the severity in the pathological progression of the disease in the EAE murine model.

## 5 DISCUSSION

In the present work, we show that LRRK2 inhibition promotes CNS remyelination in vitro and could display an essential role as well in vivo. The inhibition of LRRK2 with compound **4** promoted in vitro OPC proliferation and differentiation toward myelin‐forming phenotypes, while in vivo this compound ameliorated the clinical course of EAE demyelinating murine model. This effect was selective, because other LRRK2 inhibitors studied (the commercial PF‐475, or compounds **1**–**3** and **5**) had no effects in our experimental paradigms employed in the present work. It is also remarkable that, although the use of the effective compound **4** at 5 μM dose promoted OPC proliferation, the combined pro‐proliferation plus pro‐differentiation effect was obtained just when it was used at 1 μM concentration in the culture medium. In this condition, this LRRK2 inhibitor was as efficient as the hormone T3^41^ (a well‐known anti‐inflammatory and remyelinating agent in animal models of demyelination). Nevertheless, T3 cannot be used in MS patients because of the lack of therapeutic margin separating its benefits in the CNS from systemic thyrotoxicosis.^42^ Using a variety of animal models of disease as proofs‐of‐concept, some other pathways and drug targets have been shown to improve remyelination, to date.^43^, ^44^, ^45^ Recently, two commercially available immunomodulators have been suggested to display remyelinating activity, although the latter relies in very speculative preclinical studies.^46^, ^47^, ^48^

The effects observed here with the LRRK2 inhibitor **4** add to the debate on whether cell proliferation and differentiation are exclusive and sequential processes or if they happen simultaneously.^49^ We think that the mutual exclusion of both cellular processes makes sense in an individual cell, but not in a group of them that can be either proliferating or differentiating simultaneously, with the addition that different types of OPCs may coexist in the demyelinating scenario.^33^ Although compound **4** has not been tested in clinical trials, its remarkable (re)myelinating effects would, if confirmed in vivo and safe enough, open a new therapeutic approach for MS and other demyelinating diseases. It is also interesting to emphasize that repairing the lost myelin, even partially, is a very effective form of neuroprotection, too.^38^, ^39^, ^40^

Regarding the observed effect in EAE, our current results were relatively stronger than those obtained in other studies.^33^, ^50^, ^51^ Despite the effects directly obtained in OPCs in vitro, LRRK2 inhibition with compound **4** may reflect the anti‐inflammatory effect that is previously known from different scenarios and very recently suggested in patients with Parkinson's disease, too.^52^, ^53^ It is known that OPCs are especially vulnerable to inflammation at the peak of the disease.^54^ To which extent the observed effect of this LRRK2 inhibitor in EAE combines anti‐inflammation plus (re)myelination (as suggested by our current results in vitro) remains to be fully addressed. Thus, although we cannot discard an anti‐inflammatory effect in vivo with our current data, the cell‐based experiments strongly suggest that remyelination could be playing a role in our EAE model. The present preclinical studies would deserve additional studies in the cuprizone, LPC and or LPS demyelinating models.^55^ Although preliminary, our present data suggest that the small molecule **4** may be useful in aiding both the inflammatory T‐cell‐driven RR‐MS and the neurodegeneration observed in the progressive phase of the disease (SP‐MS, maybe the primary progressive form PP‐MS, too). If this holds true in clinical trials, then LRRK2 inhibition would become a novel beneficial therapy for MS. Treatments available for RR‐MS to date have failed to positively impact on the progressive phases of MS,^56^, ^57^, ^58^ which points to the need of future approaches combining different classes of compounds for both the RR‐MS and the progressive phases of MS (SP‐MS, PP‐MS). Although we cannot discard that compound **4** would cover both physiopathological aspects by itself, as in other compounds,^33^, ^55^ the incorporation of LRRK2 inhibitors into the MS therapeutic arsenal will open horizons for effective combinations with currently available immunomodulators. In addition, the simple chemical structure and synthesis of compound **4** opens the possibility of further structural modification to tailor its effects. These studies together with extensive pharmacological property characterization of the LRRK2 inhibitor **4** will determine whether the drug could have a better therapeutic window to be taken to clinical trials.

## 6 CONCLUSIONS

In this work, we investigated the potential of small molecule LRRK2 inhibitors, including their remyelinating characteristics for the treatment of MS. Compound **4**, which presented a 0.51 μM IC_50_ for LRRK2 inhibition, showed proliferating and differentiating properties in primary OPC cultures at micromolar concentration. These promising characteristics suggest its potential to treat demyelination in vivo, which was confirmed in the EAE murine model. In vivo results show how compound **4** reduced the clinical burden in these mice, which was associated with a significant decrease in the damaged area. Therefore, the inhibition of LRRK2 may synergistically trigger beneficial anti‐inflammatory and (re)myelination pathways with potential high impact in MS and other demyelinating diseases. Only clinical trials will confirm the real value of small molecules such as compound **4** as efficient disease‐modifying and neuroprotective therapies for these patients.

## AUTHOR CONTRIBUTIONS

Fernando de Castro, Valle Palomo, and Ana Martínez conceived and designed the research project and obtained funding to perform the experiments. Rocío Benítez‐Fernández, Fernando Josa‐Prado, Yolanda Lao, Estefanía Sánchez, Alfonso García‐Rubia, and José Cumella performed the experiments. Rocío Benítez‐Fernández, Fernando Josa‐Prado, Ana Martínez, Valle Palomo, and Fernando de Castro analyzed the data and the results. Valle Palomo, Fernando de Castro, Rocío Benítez‐Fernández, and Fernando Josa‐Prado wrote the manuscript. Ana Martínez supervised and edited the manuscript. All authors reviewed and approved the final version of the manuscript.

## CONFLICT OF INTEREST STATEMENT

The authors declare no conflicts of interest.

## DECLARATION OF TRANSPARENCY AND SCIENTIFIC RIGOR

This declaration acknowledges that this paper adheres to the principles for transparent reporting and scientific rigor of preclinical research as stated in the BJP guidelines for Natural Products Research, Design and Analysis, Immunoblotting and Immunochemistry, and Animal Experimentation, and as recommended by funding agencies, publishers and other organizations engaged in supporting research.

## Supporting information


Appendix S1.

## Data Availability

The data that support the findings of this study are available from the corresponding authors upon reasonable request.
