# Supplementary material for: Efficacy of a benzothiazole‐based LRRK2 inhibitor in oligodendrocyte precursor cells and in a murine model of multiple sclerosis
Source: CNS Neurosci Ther. 2024 Jan 24;30(1):e14552. doi: 10.1111/cns.14552 (PMC10808848; doi:10.1111/cns.14552)
Supplement: Supplementary file 1 — Appendix S1. [file CNS-30-e14552-s001.docx]

SUPPLEMENTARY INFORMATION

**Title:** EFFICACY OF A BENZOTHIAZOLE-BASED LRRK2 INHIBITOR IN OLIGODENDROCYTE PRECURSOR CELLS AND IN A MURINE MODEL OF MULTIPLE SCLEROSIS

**Authors’ Full Names:** Rocío Benítez-Fernández,^1,2,^^ Fernando Josa-Prado,^2^,^ Estefanía Sánchez, ^2^ Yolanda Lao, ^2^ Alfonso García-Rubia,^1^ José Cumella,^3^ Ana Martínez,^1,4^ Valle Palomo ^4,5,6,^*, and Fernando de Castro^2,^* ^ These authors contributed equally to this work.

**Institutional Affiliations:** ^1^Centro de Investigaciones Biológicas Margarita Salas-CSIC, Ramiro de Maeztu 9, 28040 Madrid (Spain). ^2^Instituto Cajal-CSIC, Avda. Doctor Arce 37, 28002 Madrid (Spain). ^3^Instituto de Química Médica, IQM-CSIC, Madrid, (Spain). ^4^Centro de Investigaciones Biomédicas en Red en Enfermedades Neurodegenerativas (CIBERNED), Instituto de Salud Carlos III, Av. Monforte de Lemos 3-5, 28029 Madrid (Spain). ^5^IMDEA Nanoscience, Ciudad Universitaria de Canto Blanco, C/Faraday 9, E28049 Madrid, Spain.^6^Unidad de Nanobiotecnología Asociada al Centro Nacional de Biotecnología (CNB-CSIC), 28049, Madrid, 28049 (Spain)

**Correspondence:** Fernando de Castro, MD, PhD, Instituto Cajal-CSIC, [fdecastro@cajal.csic.es](mailto:fdecastro@cajal.csic.es), + 34 915 85 46 50;

& Valle Palomo PhD, IMDEA Nanoscience [valle.palomo@imdea.org](mailto:valle.palomo@imdea.org), +34 91 299 88 49

**Table of contents.**

Scheme S1: Synthesis of compound 1……………………………………...………2

Chemical synthesis of compound 1 …………………………………………………2

LRKK2 inhibition assay of compound 1……………………………………………..4

**Scheme S1**. Chemical synthesis of compound 1. (i) 100 ºC, 2 h, MW; (ii) MeI, 25 ºC, 16 h (iii) TMSONH_2_, DMF, 160 ºC, 20 h.

**Chemical synthesis of compound 1**

Reagents were obtained from Sigma-Aldrich were used without further purification^. 1^ H NMR and ^13^C NMR data were obtained from a Bruker AV300 or AV500 MHz spectrometer. Chemical shifts, δ, expressed in parts per million (ppm) were calculated from the reference of the appropriated deuterated solvents. Signal multiplicities (s = singlet, d = doublet, dd = doublet of doublets, ddd = doublet of doublet of doublets, t = triplet, td = triplet of doublets, q = quartet, and m = multiplet) and coupling constants (*J*, Hz) are indicated for each molecule. The acquired spectroscopic data was analyzed with MestreNova 10.2 software. The microwave-assisted synthesis was carried out using a Biotage Initiator eight single-mode cavity instrument from Biotage. Experiments were performed at temperature control mode in sealed microwave vials. Temperature was measured through an IR sensor on the outside of the reaction vial. Stirring was provided by a magnetic stirrer in situ. High-performance liquid chromatography (HPLC) analyses were performed in a Thermo Finnigan Surveyor UV−vis Plus detector coupled with FinniganTM LXQ TM system. The column used for the analysis was a SunFire C18 (3.5 μm, 4.6 × 50 mm), and UV−vis spectra of the samples were acquired. Melting points were determined in a Büchi Melting Point M-560 apparatus. High-resolution mass spectrometry (HRMS) was done in an Agilent 6500 spectrometer using positive electrospray techniques (ESI). Values are expressed in mass units (m/z). The final compounds have a purit y of ≥95% as tested by HPLC.

**1-(naphthalen-1-yl)-3-(pyridin-2-yl)thiourea, Comp.6:** 1-Naphthyl isothiocyanate (2 g, 0.010 mol) was mixed with 2-aminopyridine (1.01 g, 0.010 mol) in EtOH (72 mL) and was stirred under reflux for 16 h. The solid formed (1-(naphthalen-1-yl)-3-(pyridin-2-yl)thiourea) was isolated through filtration and used for the following step without further purification. ^1^H RMN δ= ppm (300 MHz, DMSO-_d6_):13.89 (s, 1H); 11.07 (s, 1H); 8.31 (m, 1H); 7.98 (m, 2H); 7.87-7.56 (m, 6H); 7.34 (dt, *J* = 8.4 Hz, 1H); 7.12 (ddd, *J* = 7.2 Hz, *J* = 5.1 Hz, *J* = 1.0 Hz, 1H). ^13^C RMN (75 MHz, CDCl_3_): 180.1; 154.1; 145.57; 139.8; 135.2; 134.1; 129.3; 128.7; 127.0; 126.9; 126.5; 125.8; 124.8; 122.5; 118.6; 113.3. HPLC/MS (G_5-95; tg_5): t.r. = 5.13 min. m/z = 280.1 (100%), 281.1 (35%), 282.1 (10%).

**methyl (Z)-N-(naphthalen-1-yl)-N'-(pyridin-2-yl)carbamimidothioate, Comp. 7**: 1-(naphthalen-1-yl)-3-(pyridin-2-yl)thiourea (0.78 g, 0.028 mol) was dissolved in 2 mL of DMF. Methyl iodide (0.41 g, 0.0028 mol) was added to the mixture that was left stirring for 16 h at room temperature. Solvent was removed under vacuum and water and a solution of K_2_CO_3_ were added until reaching basic pH, yielding the formation of a white solid. AcOEt was added to extract the formed product and the organic phase was dried with MgSO_4_. Solvent was removed under vacuum yielding a yellow solid that was used in the next reaction without further purification. ^1^H RMN δ= ppm (300 MHz, CDCl_3_): 13.44 (s, 1H); 8.45-8.36 (m, 1H); 8.35-8.27 (m, 1H); 8.06-7.88 (m, 2H); 7.82-7.52 (m, 5H); 7.38 (d, *J* = 8.2 Hz, 1H); 7.02 (t, *J* = 6.3 Hz, 1H); 2.62 (s, 3H). ^13^C RMN (75 MHz, CDCl_3_): 162.3; 161.3; 145.2; 137.6; 134.2; 134.1; 130.2; 128.1; 127.3; 126.5; 125.1; 124.9; 122.8; 121.5; 117.3; 14.10 .HPLC/MS (G_5-95; tg_5): t.r. = 3.22 min; m/z = 294 (100%), 295 (70%).

**N-(naphthalen-1-yl)-[1,2,4]triazolo[1,5-a]pyridin-2-amine, Comp. 1**: 0.2 g (0.0006 mol) of the solid was mixed with O-(Trimethylsilyl)hydroxylamine (0.14 g, 0.0013 mol) in DMF (3 mL) in a microwave vial with activated molecular sieve. The reaction was mixed and heated at 160 ºC in a microwave for 2 h, and after that it was stirred at 200 ºC for additional 2 h. After that time, AcOET was added, the solvent was washed with water and the organic phase dried with MgSO_4_. The crude reaction was purified by flash chromatography using EtOH/Ch_2_Cl_2_/Hexane as eluent mixture (3:1:1 ratio). The fraction containing the desired product was washed with Et_2_O and the non-soluble solid was isolated as a white solid (5 mg, 3% yield). ^1^H RMN (300 MHz, CDCl_3_): 8.46 (dt, *J* = 6.7 Hz, *J* = 1.2 Hz, 1H); 8.30 (dd, *J* = 7.1 Hz, *J* = 1.6 Hz); 8.08-8.00 (m, 1H; 7.89 (dd, *J* = 7.6 Hz, *J* = 2.0 Hz, 1H); 7.67-7.40 (m, 7H); 7.30 (td, *J* = 6.7 Hz, *J*= 1.7 Hz, 1H). ^13^C RMN (126 MHz, CDCl_3_): 163.1, 150.4, 135.1, 134.31, 129.6, 128.8, 127.86, 126.3, 126.2, 125.9, 125.8, 122.7, 120.2, 115.2, 114.8, 113.2. HPLC/MS (G_5-95; tg_5): t.r. = 4.47 min; Área = 100%; m/z = 261 (100%), 262 (18%). C_16_H_12_N_4_ exact mass 260.1062:, mass found: 260.1058.

LRKK2 inhibition assay of compound **1**

LRRK2_wt_ kinase activity was measured externally following a methodology previously reported^1^. Briefly, Adapta Screen technology (Life Technologies, Invitrogen) was employed following the Adapta assay validation protocol PV4873 for LRRK2. The conversion in % of ATP into ADP by the kinase reaction was calculated for compound **1** from the ADP/ATP standard curve, and mean values were formed from each duplicate assay well. The mean conversion ratios for the kinase assay containing increasing inhibitor concentrations were fitted to a sigmoidal binding model, and the IC_50_ was determined by the intersection of the fitted curve (dose–response sigmoidal fitting) with the 50% activity. Values and standard deviations were calculated from 10 different concentrations per compound in 2 independent experiments. The LRRK2 IC_50_ value obtained for compound **1** was 3.66 ± 0.56 μM.

**Reference**

^1^ Zaldivar-Diez, J., Li, L., Garcia, A. M., Zhao, W.-N., Medina-Menendez, C., Haggarty, S. J., Gil, C., Morales, A. V, & Martinez, A. (2020). Benzothiazole-Based LRRK2 Inhibitors as Wnt Enhancers and Promoters of Oligodendrocytic Fate. *Journal of Medicinal Chemistry*, *63*(5), 2638–2655. https://doi.org/10.1021/acs.jmedchem.9b01752
